# Tracking Dietary Patterns over 20 Years from Childhood through Adolescence into Young Adulthood: The Saskatchewan Pediatric Bone Mineral Accrual Study

**DOI:** 10.3390/nu9090990

**Published:** 2017-09-08

**Authors:** Elham Z. Movassagh, Adam D. G. Baxter-Jones, Saija Kontulainen, Susan J. Whiting, Hassanali Vatanparast

**Affiliations:** 1 College of Pharmacy and Nutrition, University of Saskatchewan, Saskatoon, SK S7N 2Z4, Canada; elham.movassagh@gmail.com (E.Z.M.); sjw084@mail.usask.ca (S.J.W.); 2 College of Kinesiology, University of Saskatchewan, Saskatoon, SK S7N5B2, Canada; baxter.jones@usask.ca (A.D.G.B.-J.); saija.kontulainen@usask.ca (S.K.)

**Keywords:** dietary patterns, tracking, stability, longitudinal change, generalized estimating equations, childhood, adolescence, adulthood

## Abstract

Dietary patterns established during adolescence might play a role in adulthood disease. We examined the stability of dietary patterns (DPs) from childhood through adolescence and into young adulthood (from age 8 to 34 years). Data from 130 participants (53 females) of Saskatchewan Pediatric Bone Mineral Accrual Study (aged 8–15 years, at baseline) were included. Multiple 24-h recalls were collected annually from 1991 to 1997, 2002 to 2005, and 2010 and 2011. Using principal component analysis, “Vegetarian-style”, “Western-like”, “High-fat, high-protein”, “Mixed”, and “Snack” DPs were derived at baseline. Applied DP scores for all annual measurements were calculated using factor loading of baseline DPs and energy-adjusted food group intakes. We analyzed data using generalized estimating equations. The tracking coefficient represents correlation between baseline dietary pattern scores and all other follow-up dietary pattern scores. We found a moderate tracking for the “Vegetarian-style” (β = 0.44, *p* < 0.001) and “High-fat, high-protein” (β = 0.39, *p* < 0.001) DPs in females and “Vegetarian-style” DP (β = 0.30, *p* < 0.001) in males. The remaining DPs showed poor-to-fair tracking in both sexes. No tracking for “Western-like” DP in females was observed. Assessing overall change in DP scores from childhood to young adulthood showed an increasing trend in adherence to “Vegetarian-style” DP and decreasing trend in adherence to “High-fat, high-protein” DP by age in both sexes (*p* < 0.001), while “Western-like” and “Mixed” DP scores increased only in males (*p* < 0.001). These findings suggest that healthy dietary habits established during childhood and adolescence moderately continue into adulthood.

## 1. Introduction

Nutrition is a lifestyle factor that is associated with the etiology of numerous chronic conditions [1]. The complex mixture of nutrients and dietary components from a variety of foods, with synergistic or confounding effects on each other, might influence health outcomes [2]. In nutritional epidemiology, the dietary pattern approach has gained growing attention as an alternative to the conventional method of assessing single nutrient or food intakes [3]. Dietary patterns provide a comprehensive view and allow for assessing the contributions from various dietary aspects on health outcomes, simultaneously [4].

There is the belief that dietary habits established during childhood and adolescence might persist into adulthood. Accordingly, early modification in eating habits and behaviors might promote health, and decrease the risk of developing certain health conditions during later life [5]. However, there are limited studies evaluating the stability of dietary patterns over time from childhood to adolescence [6] or from childhood to adulthood [7]. Most studies in children and adolescents followed participants over short periods, ranging from 3 to 6 years [8,9,10,11]. Findings from these studies were inconclusive due to variations in age at baseline, length of follow-up periods, and different dietary pattern approaches or analyses used.

None of these studies [8,9,10,11] examined the consistency of dietary patterns at the individual level over the entire time. In 2003, Twisk suggested an approach to measure how individuals maintained their position in a study population distribution in subsequent measurements [12]. Using this methodology, tracking coefficients can be calculated for dietary pattern scores in longitudinal study designs with repeated measurements within individuals. The mixed longitudinal data from Saskatchewan Pediatric Bone Mineral Accrual Study (PBMAS, 1991–2011) [13,14] provides a unique opportunity for tracking dietary patterns from childhood to adulthood and overall changes in dietary pattern scores by age.

In this study, our objectives are: (1) to identify dietary patterns in the PBMAS participants at baseline; (2) to evaluate the stability of dietary patterns over the entire follow-up time and from childhood to adolescence and young adulthood; and (3) to evaluate change in dietary pattern scores by each year increase in age from 8 to 34 years of age.

## 2. Materials and Methods

### 2.1. Participants

Longitudinal data from the PBMAS participants were analyzed in this study. The details about the mixed longitudinal design of the study have been described elsewhere [13,14]. In brief, 251 Caucasian children (133 girls and 118 boys; aged 8 to 15 years) were recruited from two elementary schools in a middle-class area of Saskatoon between 1991 and 1993. All participants provided written informed consents. Participants then were followed with annual measurements until 2011 except two gaps between 1997 and 2002 and between 2005 and 2010. During each study year, anthropometrics, dietary intake, physical activity, and body composition were assessed.

For the present study, 130 participants (53 females and 77 males), with available data for 25 food group intakes, comprised our data set. There were at least five and a maximum of thirteen observations for each participant, corresponding to the total number of annual measurements conducted during the study years: 1991, 1992, 1993, 1994, 1995, 1996, 1997, 2002, 2003, 2004, 2005, 2010, and 2011. Decimal chronological age of participants was calculated using their date of birth and test date. We created chronological age groups using one-year intervals, for instance, we included observations between 8.50 and 9.49 years in the 9 years old age group. Because of varying numbers of subsequent measurements for each participant and overlaps in ages, there were a different number of observations at each age group. Overall, there was a consecutive 27-year developmental pattern (8 to 34 years) over the 20-year period of the study from 1991 to 2011. Ethics approval was obtained from the University of Saskatchewan and Royal Hospital advisory boards on ethics in human experimentation (Bio # 88-102) [13]. Written inform consent were signed by the participants before any measurement.

### 2.2. Dietary Intake

The dietary intakes of participants were assessed using 24-h recalls in the schools or in the hospital setting at the time of bone scan. For all participants, 24-h recalls were self-administered, except for the younger children from grades 2 and 3 for which the interviewer wrote down the verbally provided information. A training session on food portion sizes was conducted for children at the beginning of the study and it was reviewed before all other sessions. In addition, to determine accurate estimates of portion sizes, participants had access to life-size pictures of food items. All participants were assisted and 24-h recalls were double-checked for completeness by researchers. During the second follow-up (2010–2011), dietary intake data were collected by interviewer-administered 24-h recalls. Dietary intake information was collected three to four times per year during the first four years of study from 1991 to 1994 and two to three times per year during 1995 and 1996. Dietary intake data from multiple 24-h recalls per year were used to estimate average intake during the year. Only one 24-h recall per year was completed for each participant, thereafter. Of the total number of the participants (*n* = 251), 13 participants were excluded because of incomplete 24-h recalls. To estimate daily dietary intake of total energy and five food groups (milk and alternatives, fruit and vegetables, meat and alternatives, fat and oils, and sweets and desserts), dietary intake data collected from 1991 to 1997 was analyzed using Canadian compatible nutrition assessment software: NUTS Nutritional Assessment System, version 3.7 (Quilchena Consulting Ltd., Victoria, BC, Canada, 1988). Estimates of total energy and the five food groups intakes for the other follow-up years was obtained using Food Processor version 8.0 and its revisions (ESHA Research Inc., Salem, Ore, USA, 2003). The dietary intake data of total energy and the five food groups were available for 238 participants.

For dietary pattern analysis, quantities of consumed foods reported in the 24-h recalls were converted to gram weight. Then, we assigned all food and beverage items into 25 non-overlapping food groups based on their similarity in nutrient content or culinary usage. These food group intake data then were aligned with other measurements for each year, as annual measurements. We used the dietary intake data of 25 food groups in dietary pattern analysis. Table 1 represents the 25 food groups and food items assigned to each group. We excluded 108 participants because we did not have access to their 24-h recalls and, consequently, their dietary intake data of 25 food groups were not available for dietary pattern analysis.

### 2.3. Other Variables

Weight and stand-up height was measured following standard protocols for each participant while wearing lightweight clothing and no shoes [13]. Body mass index (BMI) was calculated as weight (kg) divided by the square of height (m^2^). Total body fat mass was measured by dual-energy X-ray absorptiometry using the Hologic 2000 Qualitative Digital Radiography (QDR) (Hologic, Inc., Waltham, MA, USA) [13,14].

Physical activity was defined as sports, games, or dance that makes you breathe hard, makes your legs feel tired, or makes you sweat. The physical activity questionnaire (PAQ) was used to assess physical activity during spare time in the previous 7 days by rating nine items in elementary schools or eight items in high schools (excluding the item regarding activity at recess) scored on a five-point scale [15]. Six of these questions were related to scaling the level of different activities in physical education classes, recess, lunch, right after school, in the evenings, and on the weekend. The other three questions were asking about the frequency of physical activity during each day, the number of hours spent watching TV, and describing the whole week activity from low to very high activity levels [16]. The average score derived from each PAQ ranged from one to five, with higher scores indicating higher levels of physical activity. To assess young adult physical activity, PAQ was modified to a seven-item questionnaire including more age-relevant activities. The school-day structure of questions was replaced with a day section structure (i.e., morning, after lunch, before supper, evening) in the PAQ for adults [16]. The PAQ was administered three times a year during first 3 years of study and two times a year thereafter. The average physical activity scores derived from PAQs collected during each year were aligned with the other annual measurements [17].

### 2.4. Statistical Analysis

Principal component analysis (PCA) was used to derive dietary patterns. We identified five dietary patterns using 25 food groups’ intake data collected at baseline from the participants in the PBMAS. The number of dietary patterns were identified by assessing the break point in the scree plot (Figure S1), eigenvalues >1.5, and proportion of variance explained by a factor. Then we ran the analysis using the five-factor solution. The orthogonal (Varimax) rotation was applied to achieve the highest interpretability.

We calculated the applied dietary pattern scores for all study years from 1991 to 2011, based on the factor loadings for the 25 food groups in the five dietary patterns (Table 2). To control for the increase in overall intake over time, first, we adjusted intakes of the 25 food groups for total energy intake (g/1000 kcal). Then, energy-adjusted food group intakes (g/1000 kcal) were multiplied by their factor loadings for each dietary pattern and were summed, or subtracted for negative loadings, to generate a dietary pattern score for each participant during each study year. This method of computing the dietary pattern scores allows food groups with higher factor loadings to have a higher contribution to the total dietary pattern score [18].

To evaluate tracking (stability) of the five dietary patterns over time (addressing the second objective), applied dietary pattern scores from 1991 to 2011 were used. For each participant, the first available measurement was considered as the baseline dietary pattern score. Dietary pattern scores for all repeated measurements were standardized for mean and standard deviation of baseline dietary pattern scores, using this formula:

$$Z-score\;\left(\mathrm{DP}\;\mathrm{score}\right)=\frac{X\left(\mathrm{DP}\;\mathrm{score}\right)-\bar{X}\left(\mathrm{baseline}\;\mathrm{DP}\;\mathrm{score}\right)}{SD\left(\mathrm{baseline}\;\mathrm{DP}\;\mathrm{score}\right)}$$

DP: dietary pattern.

Tracking coefficients for each dietary pattern were calculated using generalized estimating equations (GEE). We regressed baseline standardized dietary pattern scores (independent variable) against all other standardized dietary pattern scores (dependent variable) during the entire follow-up time, simultaneously, while adjusting for chronological age groups (age 8 to 34 years) as time-dependent variable, and sex and age at baseline as time-independent variable. As sex and its interaction with age was significant, we evaluated tracking for males and females separately. As we used the standardized values, the tracking coefficient only takes values between 0 and 1, indicating no tracking and strong tracking, respectively. However, there are no standard cutoffs indicating classification of tracking strength within this spectrum. Some investigators categorized coefficients between 0.30 and 0.60 as moderate tracking [8]. The beta coefficient for chronological age represents the change in dietary pattern score for each year of increase in age (addressing the third objective).

We averaged all the measurements collected during age 8–13 years, 14–18 years, and >18 years as childhood, adolescence, and young adulthood measurements for each participant, respectively. The number of participants who had one average measurement during childhood, adolescence, or young adulthood was 100, 119, and 124, respectively. Participants were assigned into quartiles of dietary pattern scores during childhood, adolescence, and young adulthood. Participants in quartile four had the highest adherence and those in quartile one had the lowest adherence to the corresponding dietary pattern. We compared two extreme quartiles of each dietary pattern for sex distribution by Fisher’s exact test; and for mean height, weight, fat mass, BMI, total energy intake, and physical activity score by multivariate analysis of covariate test while adjusting for age and sex, during childhood, adolescence, and adulthood, separately. Bonferroni correction was applied to account for multiple comparisons.

To assess the stability of dietary patterns we also evaluated the transition between dietary pattern score quartiles from childhood to adolescence and adulthood (addressing the second objective). The proportion of participants (%) who remained in the same quartile from childhood to adolescence or young adulthood and from adolescence to adulthood was determined and the level of agreement was estimated using the Cohen’s kappa coefficient (κ). The kappa agreement represents the proportion of participants who remained in the same quartile after accounting for the possibility of chance. Standard cut-offs for kappa coefficient are as follows: <0.2, poor; 0.2–0.4, fair; 0.41–0.6, moderate; 0.61–0.8, good; and >0.81, very good [19].

To estimate the degree of selection bias in our study, mean baseline dietary intake data available for total energy and five food groups including milk and alternatives, fruit and vegetables, meat and alternatives, fat and oils, and sweets and desserts were compared across two groups of excluded participants (*n* = 108) and included participants (*n* = 130) using multivariate analysis of variate test. Bonferroni correction was applied to account for multiple comparisons.

All statistical analysis was performed using SPSS software, version 24.0 (SPSS, Chicago, IL, USA). A *p* value < 0.05 was considered significant.

## 3. Results

We labeled the derived dietary patterns as the “Vegetarian-style” dietary pattern, rich in dark green vegetables, eggs, non-refined grains, 100% fruit juice, legumes, nuts and seeds, added fats, fruits, and low-fat milk; the “Western-like” dietary pattern, characterized by higher intakes of fruit drinks, refined grains, cream, poultry, added fats, and processed meats; the “High-fat, high-protein” dietary pattern, associated with higher intakes of high-fat milk, tomato, red meat and legumes, nuts and seeds, and lower intake of low-fat milk; the “Mixed” dietary pattern, characterized by high intake of yogurt, cheese, desserts and sweets, fish and seafood, and 100% fruit juice; and the “Snack” dietary pattern, associated with higher intakes of dressings and sauces, vegetables (excluding dark green vegetables), chips and fries, and poultry, and lower intakes of cheese (Table 2).

Tracking coefficients for standardized scores of five dietary patterns and change in the score by age from age 8 to 34 in 53 females and 77 males are presented in Table 3. The tracking coefficient represents the correlation between baseline dietary pattern scores and all other follow-up dietary pattern scores. In other words, it shows how participants maintained their position in the total sample distribution from baseline to the last follow-up measurement. The greater tracking coefficients show the higher stability of dietary patterns at the individual level. Dietary patterns ordered from more stable to less stable dietary patterns were “Vegetarian-style”, “High-fat, high-protein”, “Snack”, and “Mixed” dietary patterns, in females; and were “Vegetarian-style”, “High-fat, high-protein”, “Snack”, “Mixed”, and “Western-like” in males. The tracking coefficient for “Western-like” dietary pattern in females was not significant. Overall, we found a significant moderate tracking (0.30–0.44) for the “Vegetarian-style” and “High-fat, high-protein” dietary patterns in females and “Vegetarian-style” dietary pattern in males. The remaining dietary patterns, except the “Western-like” dietary pattern in females, showed a significant poor-to-fair tracking (0.19–0.28) in both sexes.

Changes in dietary pattern scores by age from age 8 to 34 are presented in Table 3 for 53 females and 77 males. Since all follow-up dietary pattern scores have been standardized for the baseline dietary pattern scores, the beta coefficient for the age variable represented the amount of change in *z*-score. For example, for each year of increase in age, the “Vegetarian-style” dietary pattern score increased by 0.017 *z*-score in females. Overall, the “Vegetarian-style” dietary pattern score increased and “High-fat, high-protein” dietary pattern score decreased in both females and males. In males, scores for “Western-like” and “Mixed” dietary patterns also increased by age. The “Western-like” and “Mixed” dietary patterns in females and “Snack” dietary pattern in females and males did not reveal any significant change by age (Table 3).

The mean ages of participants during childhood (aged 8–13 years), adolescence (aged 14 to 18 years), and young adulthood (aged >18 years) were 12.2 ± 0.7 years, 15.6 ± 1.1 years, and 25.1 ± 2.6 years, respectively. Table 4 presents characteristics of participants across two extreme quartiles for each dietary pattern during childhood, adolescence, and adulthood. Results showed that females had a significantly higher adherence to the “Vegetarian-style” dietary pattern and lower adherence to the “High-fat, high-protein” dietary pattern during adolescence, and a higher adherence to the “Mixed” dietary pattern during childhood and adolescence compared to males at the same age period. During childhood, higher adherence to the “Western-like” dietary pattern was associated with higher fat mass, whereas higher adherence to the “High-fat, high-protein” dietary pattern was associated with lower weight (kg), lower fat mass (kg), and lower BMI (kg/m^2^) during childhood and adolescence. During adolescence, participants in the top quartile compared to the bottom quartile of the “Vegetarian-style” dietary pattern had significantly lower total energy intake (kcal). During adulthood, higher adherence to the “Vegetarian-style” dietary pattern was associated with lower fat mass (kg). Participants who had higher adherence to the “Vegetarian-style” dietary pattern were also more physically active. Even though participants in the top quartile compared to the bottom quartile of the “Western-like” dietary pattern score had significantly lower total energy intake (kcal), higher adherence to “Western-like” dietary pattern was associated with higher fat mass during adulthood. In contrast, higher adherence to “High-fat, high-protein” was associated with higher energy intake but was not associated with fat mass during adulthood.

Table 5 shows the proportion (%) of participants who remained in the same quartile of dietary pattern score from childhood to adolescence or young adulthood and from adolescence to young adulthood. In females, the proportion ranged from 34% to 44% for “Vegetarian-style”, from 22% to 34% for “Western-like”, from 43% to 48% for “High-fat, high-protein”, from 25% to 44% for “Mixed”, and from 33% to 41% for “Snack” dietary patterns. There was a significant level of agreement in quartile assignment for “High-fat, high-protein” (κ 0.24) or “Snack” dietary patterns (κ 0.21) from childhood to adolescence; for “Vegetarian-style” (κ 0.22), “High-fat, high-protein” (κ 0.30), “Mixed” (κ 0.25), and “Snack” (κ 0.16) dietary pattern from adolescence to young adulthood, and for “Vegetarian-style” (κ 0.26) or “High-fat, high-protein” (κ 0.26) from childhood to young adulthood, in females.

In males, the proportion of participants who remained in the same quartile varied from 31% to 51% for “Vegetarian-style”, 21% to 34% for “Western-like”, 18% to 49% for “High-fat, high-protein”, 25% to 35% for “Mixed”, and 25% to 35% for “Snack” dietary patterns. There was a significant level of agreement in quartile assignment for “Vegetarian-style” (κ 0.34) or “High-fat, high-protein” (κ 0.32) from childhood to adolescence; for “Mixed” (κ 0.13) and “Snack” (κ 0.13) dietary pattern from adolescence to young adulthood, in males. No significant level of agreement was detected from childhood to adulthood in males (Table 5).

Table S1 presents the mean and standard deviation of the baseline total energy intake and five food groups intakes including milk and alternatives, fruit and vegetables, meat and alternatives, fat and oils, and sweets and desserts for two groups of excluded and included participants. The multivariate analysis of variance test with Bonferroni correction revealed no significant difference between the two groups.

## 4. Discussion

We derived five dietary patterns using the PCA method. In this method, each participant was allocated a score for each derived dietary pattern for each dietary intake measurement. Despite cluster analysis, in the PCA method we cannot categorize participants into exclusive non-overlapping groups. Hence, each participant could have a high score for more than one dietary pattern. This makes it difficult to interpret the change in scores of one dietary pattern in association with other dietary patterns. However, we could examine the stability of each dietary pattern over time, separately. We found moderate tracking by age for the “Vegetarian-style” and “High-fat, high-protein” dietary patterns in females and “Vegetarian-style” dietary pattern in males. Except for no tracking for the “Western-like” dietary pattern in females, the remaining dietary patterns showed a poor-to-fair tracking in females and males. Assessing transition between dietary pattern score quartiles from childhood to adolescence or young adulthood or adolescence to young adulthood revealed no consistent trend in males and females for different dietary patterns. Overall, the percent of participants who remained in the same quartile of scores for different dietary patterns ranged from 22% to 48% in females and 21% to 49% in males. We found an upward trend for the “Vegetarian-style” dietary pattern and a downward trend for the “High-fat, high-protein” dietary pattern in females and males and an upward trend for “Western-like” and “Mixed” dietary patterns, only in males, by age.

To our knowledge, only two studies in adults [20,21] and one study in children [9] have used GEE for tracking dietary pattern scores over time. In the China health and nutrition survey, applied scores for “traditional southern” and “modern high-wheat” dietary patterns, derived using PCA, were tracked in 9253 participants aged ≥18 years from 1991 to 2009. Dietary intake data were collected using 3-day 24-h recalls at seven time points over 18 years. Both dietary patterns were remarkably stable over time, with stronger tracking for traditional southern compared to modern high-wheat dietary patterns (0.71 vs. 0.55) [20]. In the Swedish obese subjects (SOS) study, the “energy-dense, high saturated fat and low fiber density” dietary pattern derived using the reduced-rank regression (RRR) method was tracked in 2037 severely obese subjects aged 47 ± 6 years at baseline. A semi-quantitative diet questionnaire was used to assess dietary intake at 10 time points over ten years. They found a moderate tracking of 0.40 in women and 0.38 in men [21]. Even though we used a similar approach in tracking dietary pattern scores over time, our findings are not directly comparable to these results since these studies only evaluated changes in dietary patterns during adulthood. In the Avon Longitudinal Study of Parents and Children (ALSPAC) in the UK, a “high energy, high fat, low fiber” dietary pattern identified by the RRR method in 7027 children aged 7 years at baseline were tracked 3 years and 6 years later. They reported a moderate tracking, 0.38 in girls and 0.48 in boys, from 7 to 13 years of age [9]. Despite the shorter follow-up time in this study, the tracking coefficients were comparable to those estimated in our study for the “high-protein, high fat” dietary pattern.

Another popular method of evaluating changes in dietary patterns over time is assessing the proportion of participants who remained in the same class of dietary pattern score or same cluster over time. In the Doetinchem cohort study in the Netherlands, the stability of two dietary patterns, “low-fiber bread” and “high-fiber bread”, derived using cluster analysis, was investigated in children from age 6 (*n* = 6113) to age 11 (*n* = 4916) and 16 (*n* = 4520) years. Results of the study showed that there was a good reproducibility for food groups at each cluster and almost 42% of participants remained in the same cluster of dietary patterns after 10 years [6]. In another study, changes in dietary patterns of 3823 adolescents in Brazil were investigated from age 15 to 18 years. Using latent class analysis, participants were categorized into four dietary pattern classes including “varied”, “traditional”, and “dieting” at both time points, and “processed meats” at age 15 years or “fish, fast food, and alcohol” at age 18 years. The most frequent change was the transition of participants from “processed meat” to “dieting” class (38%). However, 36% of participants in the “dieting” class at age 15 remained in the same class at age 18 years [11]. These studies were different from our study due to the shorter period of follow-up time, different dietary pattern approaches, and smaller number of repeated measurements.

Only one study has evaluated changes in dietary patterns from childhood to adulthood [7]. Mikkila et al. [7] investigated longitudinal changes of dietary patterns in participants of Cardiovascular Risk in Young Finns Study (aged 3–18 years at baseline, *n* = 1768) after 6 and 21 years using a 48-h recall at each time point. The two “traditional Finnish” and “health-conscious” dietary patterns, derived using PCA, showed a moderate correlation (0.32 and 0.38, respectively) between baseline and 21-year follow-up, with higher tracking in adolescents (aged 15–18 years) compared to children (aged 3–14 years). Assessing the transition between quintiles of dietary pattern scores showed that 41% and 38% of participants remained in the top quintile of “traditional Finnish” and “health-conscious”, respectively, after 21 years [7]. In our study, the overall proportion of participants who remained in the same quartile of the “Vegetarian-style” dietary pattern from childhood (mean age of 12.2 ± 0.7 years) to adulthood (mean age of 25.1 ± 2.6 years) was 44% in females and 32% in males. However, our findings are not directly comparable to this study because of different analytical approaches and age at baseline. A larger number of repeated measurements and the mixed longitudinal design in our study allowed for tracking dietary patterns from age 8 to 34 years with a maximum number of thirteen measurements for each participant over the entire period from childhood to adulthood.

In our study, we computed applied dietary pattern scores for all observations based on the factor loadings of the initial dietary patterns as an alternative approach to deriving dietary patterns at each time point. Several previous studies used applied dietary pattern scores similar to our study [9,20,21,22,23,24]. This method has the advantage of assessing change in a specific baseline dietary pattern over time. However, it could not identify any new dietary pattern that might have arisen during later years. For instance, in our study change in food context over the long follow-up time (20 years) was not reflected in our results. The addition of new food items such as alcohol, coffee and tea, and protein or energy drinks in one’s diet might occur as age increases. Assessing the stability of dietary patterns over time by deriving dietary patterns at each of multiple time points, especially when derived dietary patterns have altered food composition and factor loadings, is challenging.

In our descriptive analysis, we found that higher adherence to the “Vegetarian-style” dietary pattern was associated with lower energy intake and lower fat mass, while higher adherence to the “Western-like” dietary pattern was associated with higher fat mass, despite its association with lower energy intake. Higher adherence to the “High-fat, high-protein” dietary pattern was associated with lower weight, fat mass, and BMI and higher energy intake. In our previous analysis, we found a positive association between adolescent adherence to the “Vegetarian-style” dietary pattern and bone mineral content and areal bone mineral density in adolescents and adults [25].

Our findings of higher tracking for the “Vegetarian-style” dietary pattern, representing a healthy diet, and lower tracking for the “Western-like” dietary pattern, representing an unhealthy diet, are in line with other studies implying that healthy dietary habits are more stable over time [7,10,22,23,26,27]. However, some investigators reported a higher stability for the Western/unhealthy dietary pattern compared to the healthy dietary pattern [8,28]. All components of the “Vegetarian-style” dietary pattern seem to be healthy, except added fats (butter, margarine, vegetable oils, and mayonnaise). Stronger tracking or more stability means that higher or lower adherence to a “Vegetarian-style” dietary pattern that is established during childhood or adolescence could persist into adulthood. The “Western-like” dietary pattern in our study is characterized by higher intakes of fruit drinks, refined grains, cream, poultry, added fat, and processed meats. Findings from similar studies showed a similar set of components in the “Western” DP, including higher intakes of soft drinks, fried foods, meat and processed products, sweets and desserts, and refined grains. High adherence to a Western diet is associated with high intake of fat, protein, refined carbohydrates, sodium, and phosphorus [29,30]. Lower tracking for the “Western-like” dietary pattern means that adherence to the “Western-like” dietary pattern could be inconsistent over time. Therefore, it seems that childhood or adolescence is the best time for any modification in diet in terms of enhancing health conscious dietary habits. Overall, for most of the dietary patterns we found a stronger tracking in females compared to males. However, these differences are difficult to explain. In our descriptive analysis, we found that during adolescence females had a higher adherence to “Vegetarian-style” and “Mixed” dietary patterns and lower adherence to “High-fat, high-protein” dietary patterns compared to the males.

The present study has several strengths. This is the first study tracking changes in PCA-derived dietary patterns from childhood to adulthood over the entire time using GEE modeling. The stability of the dietary patterns was assessed as participants grew from their age at elementary school to early adulthood, incorporating the critical periods during growth. Owing to the mixed longitudinal design of study, it was possible to assess changes in dietary patterns from age 8 to 34 years over a 20-year period of the study. The number of available repeated measurements for each participant was more than any similar studies. We used multiple 24-h recalls representing usual intake. Compared to food frequency questionnaire, the commonly used method in other studies, this method does not depend on long-term memory and has the advantage of being more flexible and open-ended to collect more detailed food consumption data [31].

Our study has some limitations. Due to the longitudinal nature of the study, some assessment methods were changed or improved over 20 years, which made the measurements less comparable. During the first years of the study, dietary intake was assessed using self-administered 24-h recalls compared to the interviewer-administered methods that were used during the follow-up study years. However, in the self-administered method, participants were assisted by dietitians, and the completed 24-h recalls were probed by the researchers, which made it less different from the interview-administered method. In addition, participants had learned to do these on their own, over time. The validity of these methods has not been investigated in this study. However, previous studies have shown that children and adolescents can provide necessary information in self-administered 24-h recalls and that the accuracy improves by age [32]. Comparison of the two methods using web-based 24-h recall in different studies also showed that there was a good agreement for matches, intrusions, and omissions of food items and consumed serving sizes between the administration modes in both adolescents [33] and adults [34,35,36]. The other limitation is that dietary intake of participants during the study years after 1997 was assessed using only one 24-h recall per year. A single 24-h recall might be limited in capturing usual intake of participants and might affect interpretation of the higher or lower tracking observed for specific dietary patterns, especially if the components of the dietary patterns are consumed episodically. To our knowledge, there is no validation study comparing single vs. multiple 24-h recalls in terms of food group intakes.

We used NUTS Nutritional Assessment System from 1991 to 1997 and Food Processor during the study years after 1997 to estimate total energy intake. Fiber intake was calculated to have no energy contribution to the diet using NUTS software, while in Food Processor fiber is one of the contributors of total energy intake. In other words, energy intake has been underestimated and energy-adjusted amounts of food group intake and, consequently, dietary pattern scores were overestimated during the first years of study (1991–1997). Considering the definition of tracking coefficient, which is “maintenance of subjects’ position within the population distribution”, this would have only minimally influenced our analysis, because all of the study population was similarly affected by the overestimation of dietary pattern scores. In assessing overall change in the value of the dietary pattern score by each year of increase in age, despite overestimation of dietary pattern scores in the first years, we observed a significant upward trend (increased adherence) in the “Vegetarian-style” dietary pattern. Therefore, initial overestimation of “Vegetarian-style” is not in conflict with this finding. However, the observed downward trend in some dietary patterns such as the “High-fat, high-protein” dietary pattern might have been falsely induced by an overestimation of the initial years’ dietary pattern scores. Other limitations of our study were the small convenient sample size (not population-based) compared to other studies and a selection bias because of a considerable loss in number of PBMAS participants over time. We only included those participants with at least five available measurements in the analysis. Since we did not had access to the 25 food groups intake data of the excluded participants, we used five food groups intake data including milk and alternatives, fruit and vegetables, meat and alternatives, fat and oils, and sweets and desserts, and total energy intake to estimate the degree of selection bias. We found no significant difference between excluded participants’ (*n* = 108) and included participants’ (*n* = 130) five food groups and total energy intake.

## 5. Conclusions

Overall, the “Western-like” dietary pattern had the poorest tracking and the “Vegetarian-style” dietary pattern had the strongest tracking over the entire time. Tracking was stronger in females than in males and there was a fair-to-moderate tracking for “Vegetarian-style” and “High-fat, high-protein” dietary patterns. Adherence to the “Vegetarian-style” dietary pattern increased and the “High-fat, high-protein” dietary pattern decreased by age, in females and males. There was also an increase in adherence to “Western-like” and “Mixed” dietary patterns in males. This increase was independent of increases in total energy intake by age. Our findings suggest that healthy dietary habits established during childhood and adolescence could continue into adulthood. Therefore, it is necessary to implement policies for dietary intake modifications in children and adolescents to increase intake of fruit and vegetables, non-refined grains, and low-fat milk and milk alternatives, commonly regarded as the key components of a healthy dietary pattern, which could continue until adulthood.

## Figures and Tables

**Table 1:** Food groups and food items included in principal component analysis.

Food Groups	Food Items
Dark green vegetables	Asparagus, green beans, broccoli, lettuce, green pepper, sea weed, spinach, mixed greens, snow peas
Eggs	Eggs
Non-refined grains	Whole grains and partially whole grains (60%) mostly cereals, mixed granola/grain bar, cracker, oat flakes, wheat germ, whole wheat breads, puffed wheat, brown and wild rice, popcorn, barley
Fruit juice 100%	Apple juice, limeade, apple, lemon, orange juice canned or bottled, unsweetened cranberry
Legumes, nuts, and seeds	Beans (black, kidney, lima, navy, small white, soy), chickpeas, hummus, tofu, brazil nuts, coconut, almond, hazelnuts, walnuts, cashew, peanuts, mixed nuts, pecans, peanut butter, sunflower seeds
Added fats	Butter, margarine, vegetable oil, cooking oil, mayonnaise (salad dressing, miracle whip), coconut milk, dream whip, olive oil, pesto, meatless bacon bits
Fruits	All fresh and dried fruits, canned fruits (not sweetened), avocado, olives
Low-fat milk	1%, skim, rice beverage, soy beverage
Fruit drinks	Fruit juice (sweetened), fruit drinks, iced tea
Refined grains	Refined cereals, white bread, white rice, refined pasta, noodles, pop corns, ice cream cone, pie crust, pizza pop, pizza pocket
Cream	Sour cream, cream (10%, whipped or low fat)
Poultry	Chicken and turkey
Processed meats	Burger patties (beef, ham, chicken, etc.), sausages, bacon, canned meat (beef, ham, pork, chicken, turkey), dry ribs, fried chicken, nugget
High-fat milk	2%, whole, or almond milk
Tomato	Tomato (fresh, raw, cooked, canned), ketchup, clamato juice, tomato juice, pasta sauce, pizza sauce, salsa, spaghetti sauce, tomato sauce
Red meat	Beef, ham, pork, bison (ground, loin, rib, steak, stew, fried, pot roast, balls, loaf, chop)
Cheese	All kind of cheese
Yogurt	Yogurt (plain, vanilla, or fruit)
Desserts and sweets	Sweet baked products, milk desserts, jelly, chocolate, sugar, jam, syrups, honey, and candies
Fish and seafood	Fish, shrimp, lobster, mussels, pickerel, prawns, scallops
Dressings, sauces, gravy	Gravy, dressings, Caesar, French, ranch, Italian, 1000 island, Alfredo, blue cheese, chip dip, Greek, honey garlic, white sauce, sandwich spread, tartar, teen, sundried tomato
Vegetables, others	Carrots, snap beans, cabbage, cauliflower, celery, cucumber, garlic, mushroom, pepper, squash, bean sprouts, beets, onion, eggplant, radish, zucchini, potato, green peas, corn, sweet potato, and soups
Chips and fries	Potato chips, fries, corn chips, nacho, hash brown
Soft drinks	Soft drinks (sugar-sweetened or diet)
Others	Salt, spices, seasonings, additives, pickles (dill, beet), low fat sauces (mustard, hot, soy, teriyaki), vinegar

**Table 2:** Food groups included in principal component analysis and their factor loading for the identified five initial dietary patterns.

	Factor Loadings for Dietary Patterns				
	Vegetarian-Style	Western-Like	High-Fat, High-Protein	Mixed	**Snack**
Dark green vegetables	**0.64**	0.02	−0.00	0.07	−0.22
Eggs	**0.63**	−0.18	0.23	−0.05	−0.15
Non-refined grains	**0.54**	−0.13	−0.11	0.10	0.20
Added fats	**0.41**	**0.39**	−0.03	−0.04	−0.00
Fruits	**0.40**	0.24	−0.16	0.13	0.23
Others	−0.28	0.03	0.08	0.08	0.04
Fruit drinks	0.00	**0.73**	−0.04	−0.03	0.04
Refined grains	0.06	**0.66**	0.21	−0.10	−0.03
Cream	−0.06	**0.55**	−0.01	0.13	−0.02
Poultry	−0.27	**0.41**	−0.04	−0.10	**0.40**
Processed meats	−0.05	**0.35**	−0.12	0.01	−0.09
High-fat milk	−0.12	−0.17	**0.74**	−0.04	0.18
Tomato	0.22	0.30	**0.59**	−0.14	−0.34
Red meat	−0.07	−0.05	**0.52**	0.14	−0.07
Low-fat milk	**0.35**	0.03	**−0.48**	−0.01	−0.16
Legumes, nuts, and seeds	**0.45**	0.11	**0.47**	−0.09	0.06
Cheese	0.03	0.12	0.06	**0.72**	**−0.36**
Yogurt	−0.11	0.04	−0.12	**0.61**	0.19
Desserts and sweets	−0.18	−0.05	0.23	**0.59**	0.08
Fish and seafood	0.24	−0.10	−0.08	**0.52**	−0.13
Fruit juice 100%	**0.46**	0.02	−0.04	**0.49**	0.18
Dressings, sauces, gravy	0.09	−0.30	0.24	0.08	**0.64**
Vegetables, others	−0.03	0.22	0.06	−0.03	**0.58**
Chips & fries	−0.03	−0.09	−0.02	0.00	**0.40**
Soft drinks	0.00	−0.02	−0.16	−0.20	0.20
% Of variance explained	9.2	8.5	7.8	7.7	6.7

**Table 3:** Tracking coefficients of standardized dietary pattern score and change of standardized dietary pattern score by age in Pediatric Bone Mineral Accrual Study (PBMAS) participants ^a^.

	Tracking Dietary Patterns			Change in Dietary Patterns		
	β (Baseline Score)	95% CI	*p* Value	β (Age)	95% CI	*p* Value
Females (*n* = 53)						
Vegetarian-style	0.44	0.32, 0.56	<0.001	0.017	0.003, 0.031	0.020
Western-like	0.11	−0.08, 0.31	0.264	−0.009	−0.026, 0.008	0.321
High-fat, high-protein	0.39	0.27, 0.51	<0.001	−0.019	−0.031, −0.007	0.002
Mixed	0.22	0.07, 0.37	0.004	−0.014	−0.031, 0.004	0.127
Snack	0.26	0.17, 0.36	<0.001	0.009	−0.002, 0.020	0.124
Males (*n* = 77)						
Vegetarian-style	0.30	0.13, 0.46	<0.001	0.032	0.015, 0.048	<0.001
Western-like	0.19	0.07, 0.31	0.001	0.028	0.011, 0.045	0.001
High-fat, high-protein	0.28	0.15, 0.40	<0.001	−0.014	−0.027, −0.001	0.042
Mixed	0.25	0.13, 0.38	<0.001	0.019	0.003, 0.035	0.018
Snack	0.28	0.20, 0.36	<0.001	−0.013	−0.028, 0.002	0.096

**^a^** Generalized estimating equations (GEE) were used to estimate the tracking coefficient. The tracking coefficient was adjusted for chronologic age and age at baseline. In each category, *z*-scores were calculated for just the corresponding category. Beta coefficient for age represents the *z*-score change in dietary pattern score from baseline to adulthood. CI, confidence intervals.

**Table 4:** Comparing characteristics of participants across two extreme quartiles for each dietary pattern score during childhood, adolescence, and adulthood ^a^.

	Childhood		Adolescence		Adulthood	
	Quartile 1 (*n* = 25)	Quartile 4 (*n* = 25)	Quartile 1 (*n* = 30)	Quartile 4 (*n* = 30)	Quartile 1 (*n* = 31)	Quartile 4 (*n* = 31)
**Vegetarian-style**						
Sex (% Females)	28.0	52.0	26.7	73.3 *	41.9	48.4
Height (cm)	154.5 ± 6	156.0 ± 7	173.9 ± 9	171.2 ± 9	173.1 ± 9	172.7 ± 9
Weight (kg)	45.3 ± 8	46.6 ± 8	64.9 ± 12	64.4 ± 14	82.6 ± 19	73.9 ± 13
Fat mass (kg)	10.6 ± 7	11.8 ± 5	12.5 ± 6	16.7 ± 9	24.6 ± 14	18.0 ± 7 *
BMI (kg/m^2^)	18.8 ± 3	18.9 ± 2	21.3 ± 3	21.8 ± 4	27.6 ± 6	24.6 ± 3
Energy intake (kcal)	1887 ± 371	1888 ± 473	2526 ± 897	1799 ± 672 *	2651 ± 1052	2112 ± 783
PA score	3.0 ± 0.7	3.1 ± 0.4	2.5 ± 0.6	2.6 ± 0.6	2.3 ± 0.7	2.4 ± 0.5 *
**Western-like**						
Sex (% Females)	36.0	56.0	23.3	46.7	41.9	38.7
Height (cm)	155.7 ± 7.1	154.0 ± 7.0	173.6 ± 9.0	170.7 ± 11.5	174.8 ± 10.7	174.5 ± 9.7
Weight (kg)	43.8 ± 6.5	44.0 ± 7.5	64.6 ± 11.6	63.1 ± 15.0	75.0 ± 16.6	83.0 ± 18.1
Fat mass (kg)	8.85 ± 2.8	11.8 ± 5.8 *	12.0 ± 6.0	15.1 ± 9.1	19.6 ± 9.1	25.1 ± 12.8 *
BMI (kg/m^2^)	17.9 ± 1.8	18.4 ± 2.6	21.3 ± 3.1	21.5 ± 4.1	24.4 ± 4.8	27.3 ± 6.5
Energy intake (kcal)	1949 ± 407	1764 ± 439	2437 ± 760	2100 ± 1032	2585 ± 807	2192 ± 722 *
PA score	3.1 ± 0.6	2.8 ± 0.7	2.6 ± 0.5	2.3 ± 0.6	2.2 ± 0.5	2.4 ± 0.5
**High-fat, high-protein**						
Sex (% Females)	56.0	28.0	60.0	30.0 *	58.1	35.5
Height (cm)	154.8 ± 7.1	152.9 ± 5.5	169.4 ± 10.5	170.2 ± 8.3	171.3 ± 8.7	175.7 ± 9.1
Weight (kg)	47.5 ± 8.6	42.3 ± 5.8 **	64.0 ± 13.8	59.2 ± 10.5 **	74.7 ± 14.1	75.3 ± 14.0
Fat mass (kg)	13.1 ± 6.9	9.08 ± 3.8*	17.3 ± 9.7	10.9 ± 5.1 *	21.7 ± 9.4	18.1 ± 8.4
BMI (kg/m^2^)	19.6 ± 3.0	18.0 ± 2.2 *	22.2 ± 4.5	20.3 ± 2.8 *	25.5 ± 4.8	24.3 ± 3.8
Energy intake (kcal)	1831 ± 377	1768 ± 375	1919 ± 760	2339 ± 770	1894 ± 680	2595 ± 968 **
PA score	3.2 ± 0.5	3.1 ± 0.5	2.6 ± 0.5	2.5 ± 0.6	2.2 ± 0.5	2.3 ± 0.6
**Mixed**						
Sex (% Females)	20.0	64.0 **	23.3	56.7 *	25.8	41.9
Height (cm)	156.5 ± 7.7	156.3 ± 7.7	174.0 ± 10.3	169.9 ± 7.6	176.1 ± 9.0	174.9 ± 9.8
Weight (kg)	47.4 ± 6.7	49.4 ± 10.0	67.0 ± 14.0	61.9 ± 9.8	84.5 ± 16.5	78.3 ± 20.4
Fat mass (kg)	10.4 ± 4.3	15.5 ± 7.6	13.9 ± 7.7	15.4 ± 7.3	24.1 ± 12.2	20.8 ± 12.8
BMI (kg/m^2^)	19.2 ± 2.2	20.0 ± 3.7	21.9 ± 3.4	21.4 ± 2.7	27.2 ± 5.1	25.5 ± 7.0
Energy intake (kcal)	1772 ± 372	1752 ± 410	2142 ± 842	1950 ± 655	2459 ± 794	2471 ± 969
PA score	3.1 ± 0.6	3.0 ± 0.5	2.5 ± 0.6	2.6 ± 0.6	2.3 ± 0.6	2.4 ± 0.6
**Snack**						
Sex (% Females)	60.0	36.0	56.7	43.3	32.3	45.2
Height (cm)	153.8 ± 7.4	155.3 ± 6.1	170.4 ± 10.1	171.1 ± 10.1	174 ± 9.2	172.7 ± 11.1
Weight (kg)	45.5 ± 8.7	44.6 ± 6.2	64.1 ± 11.9	64.7 ± 14.0	78.1 ± 15.4	79.4 ± 16.0
Fat mass (kg)	12.3 ± 6.4	10.7 ± 4.8	15.9 ± 9.3	15.7 ± 8.1	19.6 ± 10.1	24.2 ± 14.3
BMI (kg/m^2^)	19.1 ± 3.0	18.4 ± 2.2	22.0 ± 4.1	21.9 ± 4.1	25.7 ± 5.0	26.9 ± 7.0
Energy intake (kcal)	1888 ± 460	1802 ± 363	2002 ± 852	2131 ± 772	2505 ± 983	2192 ± 706
PA score	3.0 ± 0.5	3.3 ± 0.6	2.4 ± 0.5	2.5 ± 0.6	2.2 ± 0.5	2.3 ± 0.6

^a^ Values are proportion for sex (females %) and mean ± standard deviation (SD) for continuous variables. Fisher’s exact test for proportion of females (%); Multivariate analysis of covariance for continuous variables adjusted for age and sex with Bonferroni correction for multiple comparisons were used. * Significantly different from quartile one (*p* value < 0.05), ** significantly different from quartile one (*p* value < 0.01). BMI, body mass index; PA, physical activity.

**Table 5:** Proportion (%) of participants remaining in the same quartile for dietary pattern score from childhood to young adulthood in total sample.

	Childhood vs. Adolescence			Adolescence vs. Adulthood			Childhood vs. Adulthood		
	%	Kappa Agreement	*p* Value	%	Kappa Agreement	*p* Value	%	Kappa Agreement	***p* Value**
Females	*n* = 44			*n* = 48			*n* = 45		
Vegetarian-style	34.1	0.121	0.164	41.7	0.222	0.008	44.4	0.259	0.003
Western-like	34.1	0.121	0.164	33.3	0.111	0.182	22.2	−0.038	0.663
High fat, high-protein	43.2	0.242	0.005	47.9	0.306	<0.001	44.4	0.259	0.003
Mixed	25.0	0.000	1.000	43.8	0.250	0.003	28.9	0.051	0.551
Snack	40.9	0.212	0.015	37.5	0.167	0.046	33.3	0.111	0.199
Males	*n* = 47			*n* = 65			*n* = 50		
Vegetarian-style	51.1	0.347	<0.001	30.8	0.077	0.284	32.0	0.093	0.256
Western-like	34.0	0.120	0.154	21.5	−0.046	0.517	28.0	0.039	0.629
High fat, high-protein	48.9	0.319	<0.001	24.6	−0.005	0.940	18.0	−0.094	0.250
Mixed	25.5	0.007	0.937	35.4	0.138	0.054	32.0	0.093	0.256
Snack	25.5	0.007	0.937	35.4	0.138	0.054	32.0	0.093	0.256

Childhood: 9–13 years old; adolescence: 14–18 years old; adulthood: >18 years old.

## Supplementary Materials

The following are available online at www.mdpi.com/2072-6643/9/9/990/s1, Table S1: Comparison of mean baseline total energy intake and five food groups intakes between included and excluded participants in dietary pattern analysis; Figure S1: Scree plot obtained using principal component analysis of 25 food groups to derive dietary patterns of PBMAS participants at baseline.
