# Supplementary material for: Tracking Dietary Patterns over 20 Years from Childhood through Adolescence into Young Adulthood: The Saskatchewan Pediatric Bone Mineral Accrual Study
Source: Nutrients. 2017 Sep 8;9(9):990. doi: 10.3390/nu9090990 (PMC5622750; doi:10.3390/nu9090990)
Supplement: Supplementary file 1 [file nutrients-09-00990-s001.zip › nutrients-207859-supplementary.pdf]

Supplementary File:

Table S1. Comparison of mean baseline total energy intake and five food groups intakes between included and excluded participants in dietary pattern analysis. [1]

	Included Participants (n = 130)	Excluded Participants (n = 108)	p Value [2]
Milk and alternatives (Serving/day)	2.9 ± 1.3	2.5 ± 1.4	0.052
Fruit and vegetables (Serving/day)	3.8 ± 2.2	3.8 ± 2.3	0.811
Meat and alternatives (Serving/day)	1.9 ± 1.0	1.8 ± 1.1	0.401
Fat and oil (Serving/day)	3.6 ± 2.0	4.1 ± 3.4	0.153
Sweets and desserts (Serving/day)	3.1 ± 2.0	2.8 ± 2.0	0.241
Energy intake (kJ)	7761 ± 2060	7458 ± 2771	0.336

[1] Values are mean ± SD. [2] MANOVA with Bonferroni adjustment for multiple comparisons for baseline dietary intake data of participants.

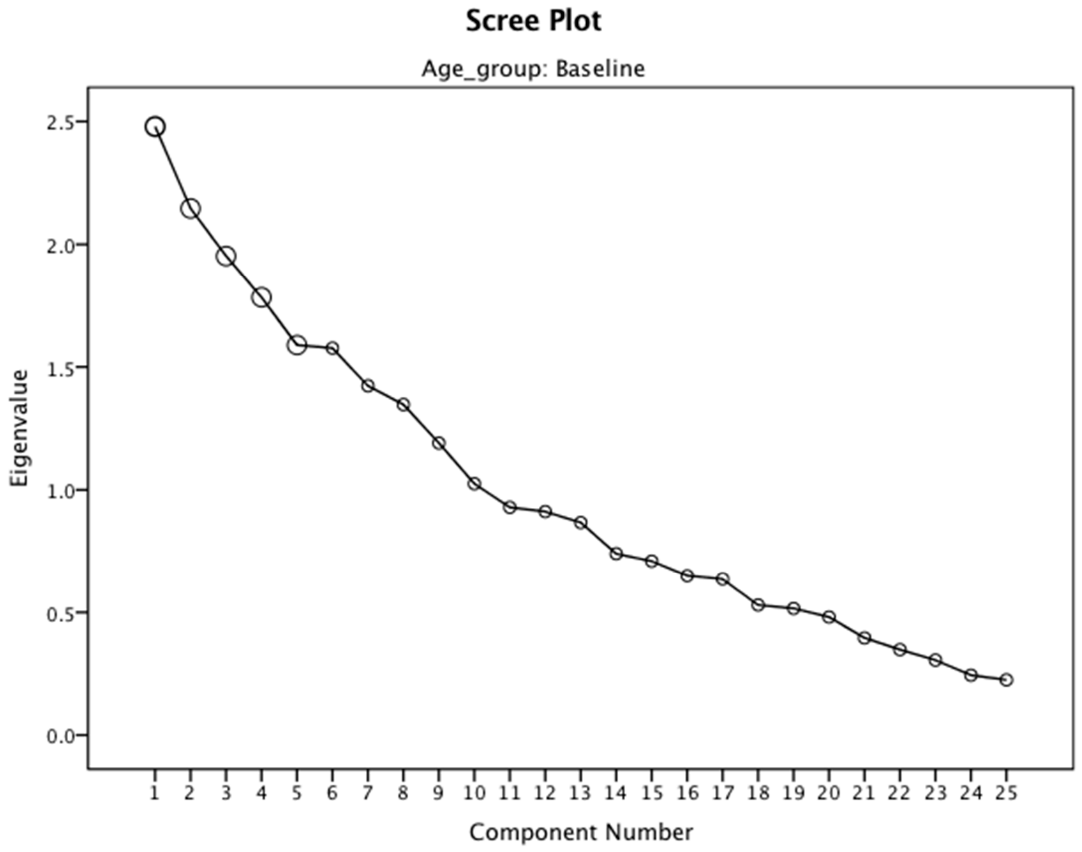

Figure S1. Scree plot obtained using principal component analysis of 25 food groups to derive dietary patterns of PBMAS participants at baseline.
